# Disinvestment in healthcare: an overview of HTA agencies and organizations activities at European level

**DOI:** 10.1186/s12913-018-2941-0

**Published:** 2018-03-01

**Authors:** G. E. Calabrò, G. La Torre, C. de Waure, P. Villari, A. Federici, W. Ricciardi, M. L. Specchia

**Affiliations:** 1 Institute of Public Health, Section of Hygiene, Catholic University of the Sacred Heart, L.go F. Vito 1, 00168 Rome, Roma Italy; 2 Department of Public Health and Infectious Diseases, Sapienza Università di Roma, Rome, Italy; 3 Department of Experimental Medicine, University of Perugia, via Gambuli 1, 06132 Perugia, Italy; 4 Italian Ministry of Health, Rome, Italy

**Keywords:** Disinvestment, Health technology assessment, Healthcare, Low-value, Disinvestment policy implementation, Resource allocation, Priority-setting, Decision-making

## Abstract

**Background:**

In an era of a growing economic pressure for all health systems, the interest for “disinvestment” in healthcare increased. In this context, evidence based approaches such as Health Technology Assessment (HTA) are needed both to invest and to disinvest in health technologies. In order to investigate the extent of application of HTA in this field, methodological projects/frameworks, case studies, dissemination initiatives on disinvestment released by HTA agencies and organizations located in Europe were searched.

**Methods:**

In July 2015, the websites of HTA agencies and organizations belonging to the European network for HTA (EUnetHTA) and the International Network of Agencies for HTA (INAHTA) were accessed and searched through the use of the term “disinvestment”. Retrieved deliverables were considered eligible if they reported methodological projects/frameworks, case studies and dissemination initiatives focused on disinvestment in healthcare.

**Results:**

62 HTA agencies/organizations were accessed and eight methodological projects/frameworks, one case study and one dissemination initiative were found starting from 2007. With respect to methodological projects/frameworks, two were delivered in Austria, one in Italy, two in Spain and three in U.K. As for the case study and the dissemination initiative, both came from U.K. The majority of deliverables were aimed at making an overview of existing disinvestment approaches and at identifying challenges in their introduction.

**Conclusions:**

Today, in a healthcare context characterized by resource scarcity and increasing service demand, “disinvestment” from low-value services and reinvestment in high-value ones is a key strategy that may be supported by HTA. The lack of evaluation of technologies in use, in particular at the end of their lifecycle, may be due to the scant availability of frameworks and guidelines for identification and assessment of obsolete technologies that was shown by our work. Although several projects were carried out in different countries, most remain constrained to the field of research. Disinvestment is a relatively new concept in HTA that could pose challenges also from a methodological point of view. To tackle these challenges, it is necessary to construct experiences at international level with the aim to develop new methodological approaches to produce and grow evidence on disinvestment policies and practices.

**Electronic supplementary material:**

The online version of this article (10.1186/s12913-018-2941-0) contains supplementary material, which is available to authorized users.

## Background

Around the world, every healthcare system is struggling with rising costs and decision makers are endeavoring to improve system efficiency and to ensure more effective and safe access to care [1]. Therefore, getting high value for patients must become a priority of healthcare delivery and this goal must belong to all stakeholders of the system. Improving health value not only will benefit patients and providers but it will result in increased economic sustainability of healthcare systems. To this aim, it is necessary to pursue a patient-centered system [2] and to foster the transformation to value-based healthcare within the organizations [3]. Therefore, a necessary goal for modern healthcare systems is to disinvest from low-value health technologies and to reinvest in high-value ones. Health technologies include different kinds of interventions (e.g. drugs, devices, medical and surgical procedures, and healthcare organizational and managerial systems) and represent a major driver of costs for healthcare systems [4].

In this value-based context and in an era of a growing economic pressure for all health systems, the interest for “disinvestment” in healthcare increased. Disinvestment is defined as “withdrawing health resources from any existing health care practices, procedures, technologies or pharmaceuticals that are deemed to deliver little or no health gain for their cost, and thus do not represent efficient health resource allocation” [5]. These actions are relevant for the sustainability of health systems because of growing demand for healthcare services, increasing availability of innovative and expensive health technologies and resource constraints. In order to tackle these challenges, evidence based approaches, such as Health Technology Assessment (HTA), are needed both to invest and to disinvest in health technologies. HTA is defined as “a multidisciplinary process that summarizes information about the medical, social, economic and ethical issues related to the use of a health technology in a systematic, transparent, unbiased, robust manner. Its aim is to inform the formulation of safe, effective, health policies that are patient focused and seek to achieve best value” [6]. HTA is an instrument supporting policy-making and is widely applied to govern the drugs market, particularly with respect to the introduction of new medicines. Anyway, in the last few years, the interest in the assessment of health technologies during their whole life cycle increased. This also led to an increasing interest into the application of HTA for disinvesting from little or no benefit technologies [7]. This interest was witnessed by the appearance of the topic of HTA for disinvestment on PubMed in the last decade even though with a low number of publications. Governmental and non-governmental agencies and organizations are entrusted to perform HTA at national and regional level across Europe and worldwide and are common forerunners of the development of innovations in the field. In order to investigate the extent of application of HTA in disinvestment at the level of HTA agencies and organizations located in Europe, methodological projects/frameworks, case studies, dissemination initiatives on disinvestment developed and released by the latter were searched.

## Methods

In July 2015, the websites of all the HTA agencies and organizations belonging to the European network for Health Technology Assessment (EUnetHTA) and the International Network of Agencies for Health Technology Assessment (INAHTA) were accessed and searched entering the term “disinvestment” in the query box. Retrieved deliverables were considered eligible if they reported methodological projects/frameworks, case studies, dissemination initiatives focused on disinvestment in healthcare and if they were published in English or Italian. Deliverables were not considered eligible if disinvestment was not the major topic or was addressed only in a narrative way. The following data were collected for each eligible deliverable: agency which released the deliverable; title of the methodological project/framework, case study, dissemination initiative; type of deliverable (i.e. position paper; methodological document; project; article; workshop); status of the deliverable (i.e. ongoing; closed); year; working group/authors; aim; field of application; methods; results and recommendations; identification of potential objectives of disinvestment. The screening process and the extraction of data were performed independently by two researchers and disagreements were solved through a consensus.

## Results

### Selection process and deliverables characteristics

A total number of 62 HTA agencies and organizations were searched. Additional file 1: Table S1 shows the list.

All the websites were accessible except for three. Out of 61 deliverables retrieved from the search, only ten met the inclusion criteria. The ten deliverables were released from 2007 onwards. They generally were relied on systematic reviews of the literature or questionnaires/interviews or data collection and analysis. The majority of deliverables were just aimed at making an overview of existing approaches and to identify challenges that might delay the introduction of disinvestment practices. Eight of them may be classified as methodological projects/frameworks [8–15], one as a case study [16] and one as a dissemination initiative [17]. All of them were closed at the time of the search except for one [10].

All the details regarding the ten deliverables are reported in the Additional file 2: Table S2.

### Methodological projects/frameworks

As for methodological projects/frameworks, two were delivered by the *Ludwig Boltzmann Institute for Health Technology Assessment* (LBI-HTA) in Austria [8, 9], one by the *Agency for Regional Healthcare* (Age.Na.S) in Italy [10], two from *Galician Agency for Health Technology Assessment* (AVALIA-T) [11] and *Agència de Qualitat i Avaluació Sanitàries de Catalunya* (AQuAS) [12] in Spain, one from the *Evaluation, Trials and Studies Coordinating Centre* (NETSCC-NIHR) [13] and two from the *Healthcare Improvement Scotland (*HIS) [14, 15] in U.K.

Deliverables released by LBI-HTA (Austria) were two systematic reviews. The first [8] was conducted in April 2011, assessing four scientific databases, websites of HTA agencies and grey literature. Policies on disinvestment from potentially obsolete technologies were the focus of the research. The aim of the review was to offer an ample overview of current disinvestment approaches of some countries (England, Spain, Australia and Canada) in order to identify the main challenges that might delay introduction of disinvestment practices in different settings and to develop recommendations for the winning achievement of disinvestment strategies. The overview of 31 articles showed that disinvestment policies in England, Spain, Australia and Canada were ongoing. The only country at a more advanced stage was Spain that had an official methodological framework – the Guideline for Not Funding existing health technologies. Instead, in England, the National Institute for Health and Care Excellence (NICE) was known issuing obligatory disinvestment advices. In Australia and Canada a dynamic debate on implementation of disinvestment policy was found, but most of the projects were yet in a piloting phase at regional level. A standard methodology of HTA for disinvestment activities was identified in all organizations studied. Nevertheless, there were not significant disinvestment projects conducted, therefore authors concluded that assessment of methods was not possible and future evidence will serve in order to evaluate whether HTA methodology for disinvestment is adequate or new methods need to be built. Furthermore, dissemination of disinvestment recommendations was considered decisive in all countries. Conversely no explicit strategies for the application of disinvestment results were proposed.

The second review of 2013 [9] was relied on five scientific databases and was aimed to identify articles describing internationally developed and implemented models for the recognition of unproductive interventions and technologies. Websites of HTA agencies, Google and relevant journals were hand searched to retrieve further information. International experts were also involved through a survey. On the basis of the results of the literature review (120 documents) and of questionnaires, eight implemented models were identified. The analysis showed that there were commonalities concerning objectives, involvement of stakeholders and choice of target groups. Most models identified ineffective interventions and technologies using person- and literature-based information sources. Effectiveness, costs and benefit were commonly applied as identification criteria. Physicians, patients and HTA-researchers were involved. Outputs – mostly HTA reports or lists – were generally distributed via internet. Recommendations were implemented either as compulsory guidelines or as non-binding information for practitioners and other stakeholders. Nevertheless, data to appraise the actual impact of models useful to identify inefficient interventions and technologies were missing.

Two HTA agencies/organizations, Age.Na.S and AVALIA-T, were involved in delivering/proposing a guide/approach to promote disinvestment. Nonetheless, the project carried out by Age.Na.S started in 2012 and was still ongoing at the time of the search, whereas the one from AVALIA-T was closed.

The Italian project was concluded in 2015 and the results were recently published [10]. The aim of the project was to develop a systematic and integrated approach to identify obsolete health technologies and to plan the implementation of new technologies in specific sectors such as: an organized screening program for HPV cervical cancer as a primary test, diagnostic imaging technologies, medical interventions and specific telemedicine applications.

The emerged crucial aspects to be considered were the following: the identification of the health needs, the obsolescence of technology, the involvement of professionals in the choices, the careful analysis of the preventive context of both investment and disinvestment and the monitoring of the effects of such choices. Concerning these aspects, the project concluded with the proposal of some concretely implementable tools represented by:

- ​​a procedure for the identification by an expert panel of interventions/procedures to be divested, starting from the systematic review of the scientific literature;

- a computerized procedure to induce professionals to identify discarded technologies according to a multi-criteria decision model;

- the methodology to analyze a regional technological park based on the assessment of the degree of obsolescence and productivity and of potential innovation;

- the methodology of ex-post evaluation of investment / renewal interventions;

- guidelines on the implementation of an innovation with the simultaneous disposal of the current alternative (i.e. the DNA-HPV test as a primary test in the Italian programs for cervical cancer screening).

The Spanish Agency AVALIA-T conducted a literature review until April 2009 in specialized databases [11], with particular attention at webpages of different national HTA agencies and government bodies, for the most part in the area of health services research. The research was focused on obsolete health technologies, including their limits and benefits. The produced guide consisted of three different sections for identification, prioritization and assessment of potentially obsolete technologies. In the first section (identification), potential sources were established: 1) direct consultation of medical literature; 2) consultation of new and rising technology databases (i.e. EuroScan); 3) consultation of systematic reviews published from scientific literature or assessment agencies; and, 4) consultation with administrative offices that are upgraded to the National Health System, hospital or regional service portfolios. After the identification, the assessment agencies would apply a standardized process to validate that the identified technology could be labeled as potentially obsolete and prioritized. In the second chapter (prioritization), a specific tool developed by AVALIA-T (PriTec tool) to prioritize potentially obsolete health technologies for following assessment was described. This tool consisted of three domains (population/end-users; risk/benefit; costs, organization and other implications) with a total of ten criteria with specific weights. Furthermore, a free of charge web application in Spanish and English was released (http://avalia-t.sergas.es/ or http://www.pritectools.es/index.php?idioma=en or www.pritectools.es) enabling up to 50 potentially obsolete health technologies to be confronted and prioritized in order to be evaluated. The final section proposed a pilot structure for the assessment of potentially outdated technologies, with a focus on the comparison of obsolete technologies with alternative ones. To assess a potentially obsolete technology, an assessment document was planned, with specific sections, focused on comparison of the benefits (in terms of efficacy, safety, efficiency, costs and other consequences) of the potentially obsolete versus the proposed alternative technology.

The second project conducted in Spain by AQuAS was published on *Health Policy* in 2013 [12]. In this paper a frame of disinvestment strategies with a “value for money” approach was proposed. Based on the experiences of other countries, it reviewed the existing instruments for implementing this approach in the Spanish National Health Service (SNS). Articulating the proposed approach to “value for money” would require three basic elements: (A) a regulatory framework, (B) the ability to identify “low value” interventions and create a guide on best practice, (C) the capacity to check compliance to and effects of “enforced” guide. Although these elements were present in the SNS from various years, their effective use in supporting a disinvestment strategy met numerous obstacles. To overcome them, the authors suggested to pool together experiences and tools and to design a process following these steps: identifying a set of “low value” procedures and their more cost-effective alternatives; mapping unjustifiable variations in the use of the selected procedures across the SNS; conforming a web-based tool for customized variability analysis to take into account excess-use and excess-costs of low value procedures in different decision-making contexts.

Three deliverables were released in U.K. [13–15]. A project was conducted by the NETSCC-NIHR in order to develop a process allowing policy-makers identifying existing procedures with uncertain appropriateness. The other two projects were conducted by HIS in 2012 and 2013 respectively. The first work was carried out by a group named *Making Choices Spending Wisely*- MaCSWise, interested in investment and disinvestment and belonging to the Scottish Health Technologies Group (SHTG). The SHTG was established by the Scottish Government Health Directorates (SGHD) and is an advisory group that aims to offer support and assistance to NHS boards when taking into consideration the introduction of new technologies to NHSScotland assessed by NICE and other HTA organizations in the U.K. and internationally. The aim of the project was to review the cost saving guidance and the ‘do not do’ recommendations released from NICE with regard to NHSScotland. Recommendations were identified that were thought to be easy to implement, likely to achieve cost savings and not requiring additional investment. Specifically, the obtained data indicated that NICE cost-saving recommendations identified from cancer guidelines were current clinical practice in the majority of NHS boards in Scotland.

The second project of HIS was undertaken to ascertain the quantity and quality of the published evidence on disinvestment. A search was carried out in the Cochrane library, NHS Economic Evaluation Database and Medline to identify relevant documents published up to July 2012. This was supplemented by general web searching, and backward and forward citation searching from included references. Articles discussing or assessing approaches used to obtain or incorporate public opinion when making any decisions relating to disinvestment were selected. Only five articles discussed frameworks, policies, processes or methods that were developed and used to obtain, consider or incorporate the views of the public during the disinvestment process. The review showed that limited research was done to discover the most suitable ways to involve the public in the decision-making process on disinvestment. The majority of articles reported that additional effort was needed to allow the public to increase and communicate informed views, and consequently to include these views during the decision-making process on disinvestment.

### Case studies and dissemination initiatives

In addition to the deliverables described, one case study and one dissemination initiative were found on the topic of disinvestment [16, 17]. The first one came from the NICE [16] whereas the second from HIS [17] in U.K. The case study was started in 2007 and it is still ongoing, while the dissemination initiative was conducted in 2013.

The “do not do recommendations” database [16] should be mentioned because it identifies and collects all recommendations about NHS clinical practices to be stopped completely or not used in daily practice. In fact, the database included more than 90 recommendations on different topics, among which: cancer care; blood and immune system conditions; cardiovascular conditions; diabetes and metabolic disorders; gastrointestinal diseases; fertility, pregnancy and childbirth; genetic diseases. Instead, the dissemination initiative was related to the organization of a session on disinvestment in a workshop realized by the SHTG team of HIS.

## Discussion

This work aimed to summarize the existing projects, frameworks, case studies and initiatives on disinvestment developed and released by HTA agencies and organizations located in Europe. Indeed, this review broadens the knowledge of disinvestment activities in Europe collating together not only projects in the field but also other types of initiatives, such as real life applications or dissemination actions. A total number of 62 HTA agencies and organizations were accessed and eight methodological projects/frameworks, one case study and one dissemination initiative were found starting from 2007.

With respect to methodological projects/frameworks, two were delivered in Austria, one in Italy, two in Spain and three in U.K. As for the case study and the dissemination initiative, both came from U.K. The majority of deliverables were aimed at making an overview of existing disinvestment approaches and to identify challenges in their introduction. Two HTA agencies/organizations, Age.Na.S and AVALIA-T, were involved in delivering/proposing a guide/approach to promote disinvestment. Age.Na.S and AVALIA-T frameworks both calls for the involvement of multiple stakeholders and for the assessment of available evidence in order to identify potential obsolete technologies. Furthermore, both frameworks rely on a multi-criteria decision analysis. Nevertheless, AVALIA-T delivered a more structured framework enclosing a tool for conducting the assessment. In particular, AVALIA-T proposed a framework for the identification, prioritization and assessment of potentially obsolete technologies used in healthcare.

Further deliverables assessed clinical practice, evaluating adherence to recommendations/guidelines and making benchmarking. In particular, one project from HIS assessed the adherence to NICE “do not do” recommendations. The case study, the “do not do recommendations” database, is to be considered as a best example identifying and collecting recommendations about clinical practices to be discontinued or not used routinely.

Despite the great interest in the field of disinvestment in healthcare, the identification of a “disinvestment framework” at an international level that could be considered a gold standard is a critical issue. Spain was the only country that had a formal methodological framework but further evidence is needed to assess its implementation and impact also outside Spain. The attention paid to the topic by the scientific community calls for the development of frameworks for disinvestment and for their real-world implementation as the main challenge.

Limited resources and increased demand for services lead healthcare systems to do choices within bound budgets underlying the need for prioritization. In fact, health systems have to allow access to effective, safe and efficient care. Priority setting may help health systems to make decisions about services to be funded or not. In this context, ‘disinvestment’ from low-value services and reinvestment in high-value ones is a key strategy which may be supported by HTA [18]. The issue of disinvestment emerged from existing HTA activities and concerns the assessment of technologies in their last phase of lifecycle. The disinvestment supported by HTA methodology started earning consideration as a policy approach for more efficient allocation of healthcare resources [8]. However, many current technologies were never assessed neither at their launch nor in their implementation phase. The lack of assessment, in particular at the end of the lifecycle, may be due also to the scant availability of frameworks and guidelines for identification and assessment of obsolete technologies that was shown by our work. Future research should address the issue of developing and testing a standardized method across European countries. In fact, although several projects were carried out in different countries, most remain constrained to the field of research. Important initiatives, for example, were conducted outside Europe, namely in Australia and New Zealand. A workshop, titled ‘Disinvestment is not a dirty word’, was held by the Health Policy Advisory Committee on Technology (HealthPACT) and supported by South Australia’s Chief Medical Officer in Brisbane on 17 May 2013. It focused on national and local disinvestment activities and the related challenges for physicians, health policy makers and funders. A report was subsequently published [19]. The following practical proposals came from this initiative: to offer advice, as appropriate, as a standing committee of the Australian Health Ministers’ Advisory Council’s Hospitals Principal Committee; to support disinvestment opportunities at national and local levels; to supply a national depository for disinvestment programs in order to guarantee pertinent information to be disclosed and to enable dialogue.

Nonetheless, detecting and eliminating potentially obsolete health technologies is a complex process that requires inputs from all relevant stakeholders, including the public and citizens, but coordinated efforts in educating all key stakeholders are necessary [18]. HTA plays a key role in favoring an evidence-based approach to pursue safety, quality, and appropriate allocation of resources and it can support the disinvestment process in healthcare [7].

Some limitations of this study need to be considered when interpreting the findings. Firstly, although the search was very wide in terms of sources, the websites of all the HTA agencies and organizations were searched using the nonspecific keyword “disinvestment”. Moreover, our search summarized methodological projects/frameworks, case studies and dissemination initiatives of HTA agencies and organizations but it excluded other works in the scientific literature or present in other databases. Hence, we found a rather small number of deliverables in line with our criteria, and this clearly led to a limited analysis of the European experiences on the field of disinvestment. Nonetheless, this study represents the first overview of HTA agencies and organizations activities trying to identify and describe the actions at European level on disinvestment in healthcare.

## Conclusion

Disinvestment is a relatively new concept in HTA that could pose challenges also from a methodological point of view. To tackle these challenges, it is necessary to go on with international experiences focused on rising novel practical approaches to generate evidence and tools for disinvestment policies and practices.

## Additional files

Additional file 1: **Table S1.** List of HTA bodies and organizations belonging to INAHTA and EUnetHTA which have been consulted (PDF 263 kb)

Additional file 2: **Table S2**. Details regarding the methodological projects/frameworks, case studies and dissemination initiatives selected (DOCX 30 kb)

## Abbreviations

Age.Na.S: Agency for Regional Healthcare
AQuAS: Agència de Qualitat i Avaluació Sanitàries de Catalunya
AVALIA-T: Galician Agency for Health Technology Assessment
EUnetHTA: European network for Health Technology Assessment
HealthPACT: Health Policy Advisory Committee on Technology
HIS: Healthcare Improvement Scotland
HTA: Health Technology Assessment
INAHTA: International Network of Agencies for Health Technology Assessment
LBI-HTA: Ludwig Boltzmann Institute for Health Technology Assessment
MaCSWise: Making Choices Spending Wisely
NETSCC-NIHR: Evaluation, Trials and Studies Coordinating Centre
NICE: National Institute for Health and Care Excellence
SGHD: Scottish Government Health Directorates
SHTG: Scottish Health Technologies Group
SNS: Spanish National Health Service
