# Supplementary material for: Disinvestment in healthcare: an overview of HTA agencies and organizations activities at European level
Source: BMC Health Serv Res. 2018 Mar 1;18:148. doi: 10.1186/s12913-018-2941-0 (PMC5831213; doi:10.1186/s12913-018-2941-0)
Supplement: Supplementary file 1 — Table S1. List of HTA bodies and organizations belonging to INAHTA and EUnetHTA which have been consulted (PDF 263 kb) [file 12913_2018_2941_MOESM1_ESM.pdf]

Table 1. List of HTA bodies and organizations belonging to INAHTA and EUnetHTA which have been consulted

Country	HTA agency	INAHTA	EUNETHTA
AUSTRIA	GÖG– Gesunheit Österreich GmbH	x	x
	LBI-HTA– Ludwig Boltzmann Institute for Health Technology Assessment	x	x
	HVB - Hauptverband der Österreichischen Sozialversicherungsträger (Association of Austrian Social Insurance Institutions)		x
BELGIUM	KCE– Belgian Health Care Knowledge Centre	x	x
BULGARIA	NCPHP - National Center of Public Health Protection		x
	NCPRMP - National Council for Pricing and Reimbursement of the Medicinal Products		x
CROATIA	AAZ - Agency for Quality and Accreditation in Health Care and Social Welfare		x
CYPRUS	MoH Cyprus - Ministry of Health of Cyprus		x
CZECH REPUBLIC	MoH Cz Rep - Ministry of Health of the Czech Republic*		x
DENMARK	HTA-HSR/DHTA– HTA & Health Services Research*	x	
	DHMA - Danish Health and Medicines Authority		x
	CFK - HTA and Health Services Research, Public Health and Quality Improvement, Central Denmark Region		x
ESTONIA	UTA - Department of Public Health of the University of Tartu		
FINLAND	FinOHTA- Finnish Office for Health Technology Assessment	x	
	FIMEA - Finnish Medicines Agency		x
	THL - National Institute for Health and Welfare		x
FRANCE	CEDIT- Comité d´Evaluation et de Diffusion des Innovations Technologiques	x	
	HAS– Haute Autorité de Santé	x	x
GERMANY	DAHTA @ DIMDI- German Agency for HTA at the German Institute for Medical Documentation and Information	x	x
	G-BA– The Federal Joint Committee (Gemeinsamer Bundesausschuss)	x	
	IQWiG– Institut für Qualität und Wirtschaftlichkeit im Gesundheitswesen	x	x
GREECE	NSPH - National School of Public Health		x
HUNGARY	OGYÈI - National Institute of Pharmacy and Nutrition		x
IRELAND	HIQA– Health Information and Quality Authority	x	x
ITALY	Agenas- The Agency for Regional Healthcare	x	x
	ASSR– Agenzia Sanitaria e Sociale Regionale (Regional Agency for Health and Social Care)	x	x
	UVT– HTA Unit in A. Gemelli Teaching Hospital	x	x
	AIFA - Italian Medicines Agency		x
	Regione del Veneto		x
LATVIA	NHS - National Health Service, NHS		x
LITHUANIA	VASPVT– State Health Care Accreditation Agency under the Ministry of Health of the Republic of Lithuania	x	
	SMCA - State Medicines Control Agency under the Ministry of Health of the Republic of Lithuania		x
LUXEMBOURG	CEM– Inspection générale de la sécurité sociale (IGSS) Cellule d'expertise médicale	x	
	CMSS - Administration du Contrôle Médical de la Sécurité Sociale		x
MALTA	MEH - Directorate for Pharmaceutical Affairs, Ministry for Energy and Health		x
NORWAY	NOKC– Norwegian Knowledge Centre for the Health Services	x	x

POLAND	AHTAPol– Agency for Health Technology Assessment in Poland	x	
	AOTMiT - Agency for Health Technology Assessment and Tariff System		x
PORTUGAL	INFARMED - National Authority of Medicines and Health Products		x
ROMANIA	NSPH MPD - National School of Public Health, Management and Professional Development		x
SLOVAKIA	MoH Slovak Republic - Ministry of Health of the Slovak Republic		x
SLOVENIA	IER - Institute for Economic Research		x
	NIJZ - National Institute of Public Health of the Republic of Slovenia		x
SPAIN	AETS - Agencia de Evaluación de Tecnologias Sanitarias	x	x
	AETSA- Andalusian Agency for Health Technology Assessment	x	
	AQuAS– Agència de Qualitat i Avaluació Sanitàries de Catalunya	x	x
	AVALIA-T– Galician Agency for Health Technology Assessment	x	x
	OSTEBA– Basque Office for Health Technology Assessment*	x	x
	IACS- Aragon Health Sciences Institute		x
	ISC III - Instituto De Salud Carlos III		x
	SESCS - Evaluation AND Planning Unit - Directorate of the Canary Islands Health Service		x
	UETS - Health Technology Assessments Unit		x
SWEDEN	SBU– Swedish Council on Technology Assessment in Health Care	x	
SWITZERLAND	MTU-SFOPH– Medical Technology Unit – Swiss Federal Office of Public Health	x	
	SNHTA - Swiss Network for HTA		x
THE NETHERLANDS	ZonMw– The Netherlands Organisation for Health Research and Development	x	
	ZIN– Zorginstituut Nederland	x	x
UNITED KINGDOM	CRD– Centre for Reviews and Dissemination	x	
	HIS – Healthcare Improvement Scotland	x	
	NIHR– National Institute for Health Research	x	
	NETSCC - NIHR, Evaluation, Trials and Studies Coordinating Centre, NETSCC		x
	NICE - The National Institute for Health and Care Excellence		x

*: website was not accessibile
