# Supplementary material for: Disinvestment in healthcare: an overview of HTA agencies and organizations activities at European level
Source: BMC Health Serv Res. 2018 Mar 1;18:148. doi: 10.1186/s12913-018-2941-0 (PMC5831213; doi:10.1186/s12913-018-2941-0)
Supplement: Supplementary file 2 — Table S2. Details regarding the methodological projects/frameworks, case studies and dissemination initiatives selected (DOCX 30 kb) [file 12913_2018_2941_MOESM2_ESM.docx]

Table 2. Details regarding the methodological projects/frameworks, case studies and dissemination initiatives selected

| **Agency** | **Title** | **Deliverable** | **Status of the deliverable** | **Year** | **Working group/authors** | **Aim** | **Field of application** | **Methods** | **Results and recommendations** | **Identification of potential objectives of disinvestment** | **Classification:**  **-methodological project/framework,**  **-case study**  **-dissemination initiative** |
| --- | --- | --- | --- | --- | --- | --- | --- | --- | --- | --- | --- |
| **LBI-HTA-AUSTRIA** Ludwig Boltzmann Institute for Health Technology Assessment | Disinvestment. Overview of disinvestment experiences and challenges in selected countries | Project report | Closed | 2011 | Gerdvilaite, J. and Nachtnebel, A. | To provide a comprehensive overview of existing disinvestment approaches in order to identify common challenges which might hamper introduction of disinvestment activities in other settings and to derive recommendations for the successful implementation of disinvestment strategies | Research | Systematic literature review. Selection criteria were policy perspectives with a focus on disinvesting in obsolete or potentially obsolete technologies | The overview shows that disinvestment policies in England, Spain, Australia and Canada are at the developing/piloting phase. Only Spain has a formal methodological framework. The National Institute for Health and Clinical Excellence in England is recognized as already issuing mandatory disinvestment advice. Active discussion towards implementation of disinvestment policy was found in Canada and Australia, but actual projects are still in the piloting phase at regional level | No | methodological project/  framework |
| **LBI-HTA-AUSTRIA** Ludwig Boltzmann Institute for Health Technology Assessment | Identification of ineffective interventions and technologies: existing models and their implementation | Project report | Closed | 2013 | Mayer, J. and Nachtnebel, A. | To provide a comprehensive overview of existing approaches for the identification of ineffective interventions and technologies | Research | Systematic literature review to identify articles describing internationally developed and implemented models for the identification of ineffective interventions and technologies. International experts were consulted via a half-standardised questionnaire | Eight implemented models were identified. Most models indentified ineffective interventions and technologies using person- and literature-based information sources. Frequently applied identification criteria were effectiveness, costs and benefit. Outputs – mostly HTA reports or lists – were generally disseminated passively via internet. Recommendations were implemented either as binding guidelines or as non-binding information for physicians and other stakeholders. Based on the models analysed, a potential model for the Austrian context has been developed. Currently, there is not enough data to appraise the actual impact of models for the identification of ineffective interventions and technologies. | No | methodological project/  framework |
| **Italy-Agenas** Agenzia Nazionale per i Servizi Sanitari Regionali | Methods for investments/ disinvestments and distribution of health technologies in Italian Regions | Project | Ongoing at the time of the search – currently closed | 2012-2013 | Chief Investigator: Marina Cerbo (Age.Na.S)  Units involved: • ARSS Agenzia Regionale Socio Sanitaria del Veneto • A.Re.S. Puglia - Agenzia Regionale Sanitaria Regione Puglia • Regione Umbria • Regione Siciliana • Azienda Ospedaliero Universitaria San Giovanni Battista di Torino • Direzione Generale Sanità Regione Lombardia • Regione Calabria • Agenzia Sanitaria Regionale Abruzzo - ASR Abruzzo • Laziosanità - Agenzia di Sanità Pubblica, Regione Lazio • Agenzia Sanitaria e Sociale Regionale - Regione Emilia Romagna • Provincia Autonoma di Bolzano | To develop an integrated and systematic approach to identify obsolete health technologies and to plan the deployment of new technologies in specific areas. | • screening program, particularly the HPV test in primary screening; • Imaging and other diagnostic technologies; • Procedures: medical interventions and specific applications of telemedicine and telecare | The different units involved in this project will contribute to the development of the methodology focusing on the use, in different regional contexts, of different health technologies | Proposal of some concretely implementable tools represented by:  - a procedure for the identification by an expert panel of interventions/procedures to be divested, starting from the systematic review of the scientific literature;  - a computerized procedure to induce professionals to identify discarded technologies according to a multi-criteria decision model  - the methodology to analyze a regional technological park based on the assessment of the degree of obsolescence and productivity and of potential innovation  - the methodology of ex-post evaluation of investment / renewal interventions  - guidelines on the implementation of an innovation with the simultaneous disposal of the current alternative (i.e. the DNA-HPV test as a primary test in the Italian programs for cervical cancer screening). | Yes | methodological project/  framework |
| **AVALIA-t- SPAIN** Galician Agency for HTA | Identification, prioritisation and assessment of obselete health technologies | Project | Closed | 2007 | Ruano Raviña A, Velasco González M, Varela Lema, Cerdá Mota T, Ibargoyen Roteta N, Gutiérrez Ibarluzea I, et al. | To produce a guide to promote the necessary basis to identify, prioritize and assess health technologies in use. | Research | Literature review. Records were selected if dealing with any type of obsolete technology or opinions, ideas, advantages or limitations concerning any aspect linked to obsolete health technologies | The project has led to: a) the identification of an evaluation framework consisting of three phases (identification, prioritisation and assessment of potentially obsolete health technologies); b) the release of an assessment-document structure with different sections centred on comparison of the benefits (in terms of efficacy and safety, efficiency, costs or other implications) of the potentially obsolete versus the proposed alternative technology. | No | methodological project/  framework |
| **AQuAS–** Agència de Qualitat i Avaluació Sanitàries de Catalunya | Disinvestment in the age of cost-cutting sound and fury. Tools for the Spanish National Health System | Paper (Health Policy 110 (2013) 180– 185) | Closed | 2013 | Sandra García-Armestoa, Carlos Campillo-Artero, Enrique Bernal-Delgadoa | This paper proposes the framing of disinvestment strategies as the “value for money” approach suitable for the current situation of acute budget restrictions. | Research | Literature review and analysis of the political and economic context | Building on the experiences from other countries, it first reviews the instruments already available for implementing the disinvestment approach within the Spanish National Health Service (SNS). The strategy for disinvestment should seek synergies between the National Network of HTA Agencies and the Atlas VPM network. A stepwise process can be designed following these broad lines: 1. Identifying a set of procedures considered low value care and their more cost-effective alternatives. 2. Mapping out unwarranted variations in the utilization rates of the procedures in the list across the SNS. 3. Adapting the Atlas VPM web-based tool for tailored variability analysis to include excess-utilization and excess-costs analysis for each of the low value procedures and in different decision-making scenarios. | No | methodological project/  framework |
| **United Kingdom- NETSCC NIHR, Evaluation, Trials and Studies Coordinating Centre, NETSCC** | Using clinical practice variations as a method for commissioners and clinicians to identify and prioritise opportunities for disinvestment in health care: a cross-sectional study,  systematic reviews and qualitative study | Project | Closed | Start date April 2011 Publication date April 2015 | Chief Investigator Professor William Hollingworth  Co-investigators Dr Padmanabhan Badrinath, Professor Jenny Donovan (University of Bristol), Dr Christine Hine, Deborah Lee (NHS Bristol), Dr Amanda Owen-Smith (University of Bristol), Professor Jonathan Sterne (University of Bristol), Louise Tranmer (NHS Bristol), Dr Penny Whiting (University of Bristol) | To develop the processes by which NHS policy-makers may identify existing procedures where there is uncertainty about appropriate use and by which local commissioners may identify procedures that might be overutilised and are potential candidates for disinvestment. Specific objectives: 1. Use routine inpatient data to identify procedures with the highest inter-Primary Care Trust (PCT) variation in use and explore whether or not high variation is a marker of clinical uncertainty  2. Work with two PCT commissioning groups to use benchmarking to select two procedures that might be locally overutilised 3. Conduct rapid systematic reviews for these two procedures to summarise the (cost-) effectiveness evidence. Discuss the possible causes of high local utilisation and options for regulating procedure use to achieve disinvestment 4. Understand obstacles and solutions to local commissioners achieving disinvestment and explore patient's and surgeon's perspectives on regulating access to secondary care procedures | Research | (1) Consultation of Hospital Episode Statistics admitted patient care data set to identify commonly used procedures between 2007/8 and 2011/12. (2) Benchmarking of local procedure rates against the national rates. (3) Rapid systematic reviews of topics selected by PCTs in order to identify systematic reviews and randomised controlled trials.  (4) Non-participant overt observations of PCT commissioning group meetings and semistructured interviews with individuals affiliated with these groups, complemented by document analysis of policies and meeting minutes/agendas. (5) Case study of disinvestment. | (1) On the basis of the analysis of 154 procedures with high geographical variation, it emerged that five factors were potentially associated with high geographical variation: (1) coding uncertainty; (2) variation in community care; (3) uncertainty about the appropriate setting; (4) urgency and invasiveness of the procedure; and (5) evolving or uncertain evidence.  (2) Two PCT commissioning groups (PCT1 and PCT2) were considered. Carpal Tunnel Release (CTR) surgery was selected in PCT1. Laser capsulotomy was selected in PCT2. In both PCTs a large number of procedures had a utilisation rate much greater than the national average.   (3) Six RCTs compared surgery with a non-surgical intervention in Carpal Tunnel Syndrome (CTS). In particular: 3 trials concluded that surgery was more likely to have a successful outcome; 2 trials with longer follow-up found the effect of surgery diminished over time, but was still evident at 12 months; one RCT estimated the cost per quality-adjusted life-year was £285, suggesting that surgery was a cost-effective intervention. Systematic reviews or RCTs of interventions for posterior capsule opacification were not found. National guidance on referral or treatment thresholds for capsulotomy were not found.  (4) Perceived barriers to disinvestment included lack of collaboration between commissioners and providers, lack of central support for disinvestment, lack of disinvestment tools and a culture of discomfort in health care about explicit discussion of costs.   (5) A case study of disinvestment (CTR surgery) was conducted. Clinicians felt threshold criteria had little impact on their practice and that prior approval systems for regulating procedure rates would not prove cost-effective. Most patients were not aware of rationing. Generally, patients interpreted threshold policies as a fair and efficient approach to control access to CTS surgery. Thresholds were acceptable to patients because of the potential of avoiding surgery and the perceived low priority of CTS. | No | methodological project/  framework |
| **HIS – Healthcare Improvement Scotland** | MaCSWise - Making Choices Spending Wisely- A report of current practice in NHSScotland relating to National Institue of Health and Clinical Excellence cost-saving recommendations | Project | Closed | 2012 | MaCSWise group. It had two main aims: 1. To devise a method of auditing whether or not NICE cost-saving and "do not do" guidance was current practice in NHSScotland;  2. To pilot the audit method using guidance identified as being of significant importance for NHSSscotland | To review the cost saving guidance and ‘do not do’ recommendations from the National Institute for Health and Clinical Excellence (NICE) with regard to NHSScotland. | Research | Recommendations were identified that were thought to be easy to implement, likely to achieve cost savings and would not require additional investment. In order to investigate the implementation of the identified recommendations, a questionnaire was developed to ascertain current clinical practice and opinion in relation to them. Data were also obtained from the Information and Statistics Division (ISD) of NHS National Services Scotland | The data indicate that NICE cost-saving recommendations identified from cancer guidelines are current clinical practice in the majority of NHS boards in Scotland. The questionnaire responses suggest greater variation across NHSScotland in practice relating to recommendations from guidelines identified for other therapeutic areas. ISD data suggest that the following NICE "do not do" recommendations may merit further investigation to realise potential cost savings in Scotland: arthroscopic knee washouts for osteoarthritis; laparoscopic uterine nerve ablation for pelvic pain; and corneal implants for refractive error. | Yes | methodological project/  framework |
| **HIS – Healthcare Improvement Scotland** | What approaches have been taken and efforts made to ensure public involvement in decision making relating to potential disinvestment in healthcare interventions and technologies? | Technologies scoping report | Closed | 2013 | Healthcare Improvement Scotland development; Members of the SHTG evidence review committee team | This scoping report was undertaken to ascertain the quantity and quality of the published evidence on this topic | Research | Literature review. Articles discussing or assessing approaches used to obtain or incorporate public opinion when making any decisions relating to disinvestment were selected. | This review shows that little research has been done to find the best ways of involving the public in making healthcare decisions as regards to disinvestment. There was no strong evidence to suggest that any of the frameworks identified could be considered a best practice model. Most of the articles reported that more effort is needed to enable the public to develop and express informed views, and consequently to incorporate these views when making disinvestment decisions. There is limited evidence that specifically answers the question on how public views have been integrated into decisions relating to the disinvestment of healthcare services. Thus, it has been difficult to identify any suitable approaches for NHSScotland. | No | methodological project/  framework |
| **NICE- NHS- National Institute for Health and Clinical Excellence** | Do not Do Recommendations | Recommendations database | Ongoing | Since 2007 | NICE team | To identify and collect into a database all recommendations about NHS clinical practices to be discontinued completely or not used routinely. | Clinical practice | Recommendations are abstracted from NICE cancer service guidance, clinical guidelines, interventional procedures and technology appraisals guidance and updated or replaced as new guidance is published. Each record contains the 'do not do' recommendation and includes additional information including the intervention, health topic, the guidance it comes from (with a link to the relevant paragraph in the guidance) and the other 'do not do' recommendations from the same guidance. | More than 90 "Do not Do" recommendations on different topics, among which: cancer care, blood and immune system conditions; cardiovascular conditions; diabetes and metabolic disorders; gastrointestinal diseases; fertility, pregnancy and childbirth; genetic diseases, etc. | Yes | Case study |
| **HIS – Healthcare Improvement Scotland** | SHTG topic generation workshop | Workshop | Closed | 2013 | SHTG team | Key objectives of the workshop were to: • Enable members, the secretariat and technical staff to come together and discuss issues related to the group’s functions and future development. • Reflect on the SHTG work programme and outputs to date and consider whether efforts are adding most value for NHSScotland. • Highlight international approaches to topic generation and prioritisation. • Consider whether to develop a proactive element to the SHTG work programme and how to identify materially significant topics, including disinvestment topics, for SHTG to consider. |  | It was organized a session on disinvestment |  | No | Dissemination initiative |
